# Vascularized Fibula for Early Management of Upper Extremity Trauma With Critical Bone Loss: A Report of Two Cases

**DOI:** 10.1155/cro/2860926

**Published:** 2026-10-28

**Authors:** Brian Chen, Lindsey Herrod, Mayo Hotta, Jorge L. Rocha, Martin I. Newman, James W. Fletcher

**Affiliations:** ^1^ Department of Plastic Surgery Cleveland Clinic Florida Weston Florida USA clevelandclinic.org; ^2^ Department of Plastic Surgery Broward Health Medical Center Fort Lauderdale Florida USA browardhealth.org; ^3^ Department of Orthopedic Surgery Broward Health Medical Center Fort Lauderdale Florida USA browardhealth.org; ^4^ Vanguard Plastic Surgery Fort Lauderdale Florida USA

**Keywords:** bone loss, case report, free flap, microsurgery, trauma, upper extremity, vascularized fibula

## Abstract

**Introduction:**

We present two Type 3B open fracture cases with critical bone loss in the upper extremity: a 9 cm ulnar defect and 8.5 cm distal humerus defect treated with vascularized fibula graft 6 and 21 days postinjury, respectively. Both patients had complete healing of soft tissues, no known complications, and returned to heavy duty labor within 3 and 5 months of injury. Although the use of vascularized fibula graft is well described, the novelty of our cases lies in its use as the initial attempt at definitive fixation of acute upper extremity trauma with critical bone loss, rather than as a salvage procedure following nonunion or infection.

**Conclusion:**

Critical bone loss following upper extremity trauma is rare and challenging. The vascularized fibula graft is ideal for these cases and should be considered for acute, single‐stage reconstruction of traumatic upper extremity bone defects to achieve maximal outcomes in the shortest time.

## 1. Introduction

Critical bone loss impairs the body′s inherent ability to heal despite surgical stabilization [1] and is typically managed with induced‐membrane formation [2] or distraction osteogenesis/bone transport; [3] but vascularized bone grafting is a powerful reconstructive option as it provides a stable scaffold with osteoconductive, osteoinductive, and osteogenic properties to achieve union in a single‐stage [4–8]. Among donor options, the vascularized fibular graft (VFG) is optimal for long bone defects, particularly when there is need for simultaneous bone and soft tissue reconstruction. Despite this, it is mostly used as salvage for nonunion, infections, or for oncologic reconstruction [6, 7, 9].

Critical bone loss after trauma afflicts the lower extremities in 90% of cases [1], so fewer studies report upper extremity traumatic reconstruction with VFG [5, 10, 11]. In these, reconstruction occurs in a delayed fashion with the earliest more than a month after injury [5, 11, 12].

The VFG is underutilized as acute treatment for traumatic critical bone loss in the upper extremity, possibly because of fear of the zone of injury which may risk flap failure, though this has been disproved [13, 14]. It may also be viewed as a salvage procedure because of its need for microvascular expertise and donor site morbidity [1, 4].

We report on two cases of reconstruction of upper extremity trauma with critical bone loss utilizing VFG 6 days and 21 days after inciting trauma. CARE guidelines were adhered to. All operations were performed at a single level 1 trauma center by orthopedic traumatologists and reconstructive microsurgeons. Although this technique is being more widely applied in limb reconstruction, the novelty of these reports lies in its use as the initial attempt at definitive fixation in this challenging subset of patients.

### 1.1. Consent

Both patients gave written consent for publication of their cases and images.

### 1.2. Technique

VFG harvest begins with designing a skin paddle in the distal third of the fibula based on adequate perforators. Under tourniquet, lateral compartment muscles are reflected anteriorly and the posterior compartment entered through the intermuscular septum. Flexor hallucis longus is elevated off the fibula taking care to protect the peroneal nerve and anterior tibial vessels. Osteotomies are performed proximally and distally leaving 4 cm at the knee and 6 cm at the ankle for joint stability. The peroneal pedicle is circumferentially dissected and prepared for microsurgery.

## 2. Case Presentations

### 2.1. Case 1

A 26‐year‐old male with no significant medical or surgical history sustained an open left ulna shaft fracture from self‐inflicted ballistic injury (Figure 1). He presented with paresthesias in the ulnar distribution as well as 3/5 intrinsic strength, but had a palpable radial pulse and well perfused hand. He went to the operating room (OR) on hospital Day 1 revealing ulnar artery transection, contused but intact ulnar nerve, a 12 × 5 cm soft tissue defect, and severely comminuted, devitalized bone devoid of periosteum leading to a 9 cm bone defect (Figure 2). On Day 6, underwent reconstruction with VFG with a 6 x 10 cm skin paddle.

**Figure 1:**
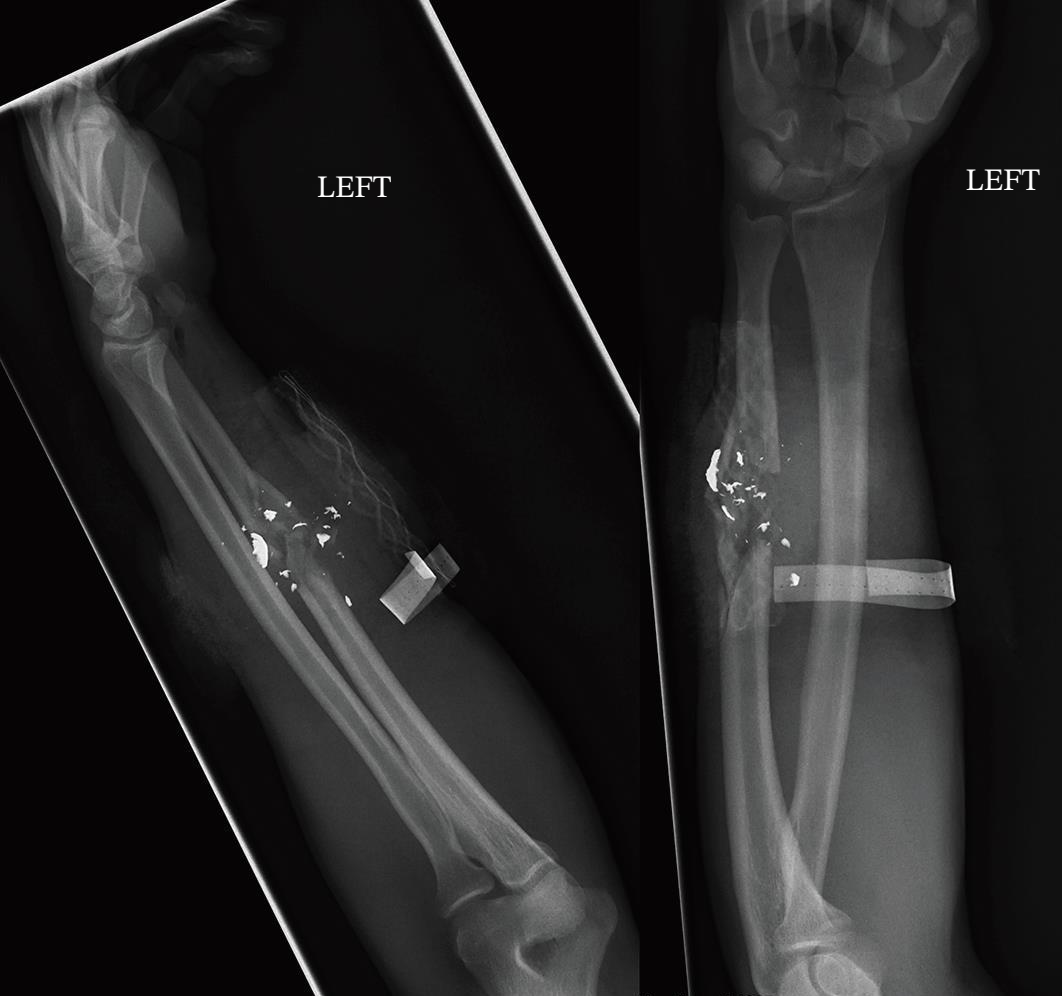
Open left ulnar shaft fracture from ballistic injury with comminution and devitalized bone.

**Figure 2:**
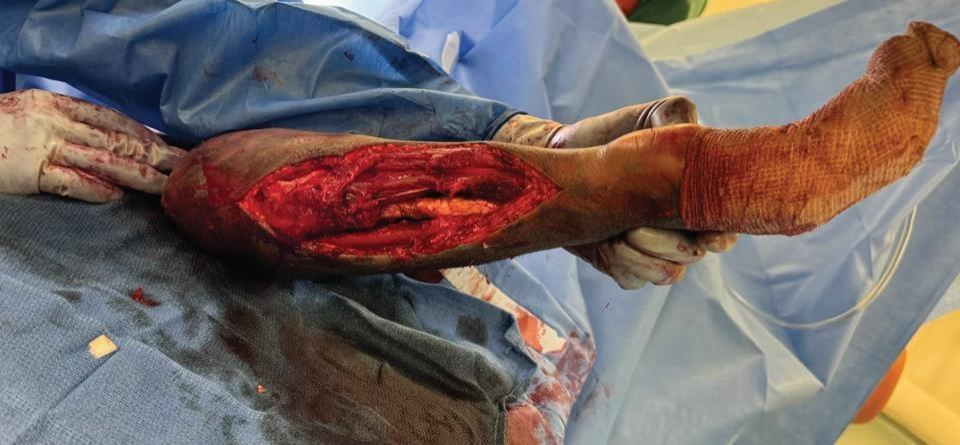
Open left ulnar shaft fracture with extensive soft tissue injury and 9 cm bone defect, here pictured with antibiotic cement.

The peroneal pedicle was anastomosed to the ulnar vessels proximal to level of traumatic transection. An external distractor was placed to open the ulnar defect and the fibula placed as an interposition graft. An antegrade flexible nail was placed into the ulna and VFG, followed by compression through the distractor until the distal radioulnar joint was neutral with smooth pronosupination (Figure 3). Fluoroscopy confirmed adequate length, rotation, and alignment whereas the skin paddle achieved excellent soft tissue coverage (Figure 4).

**Figure 3:**
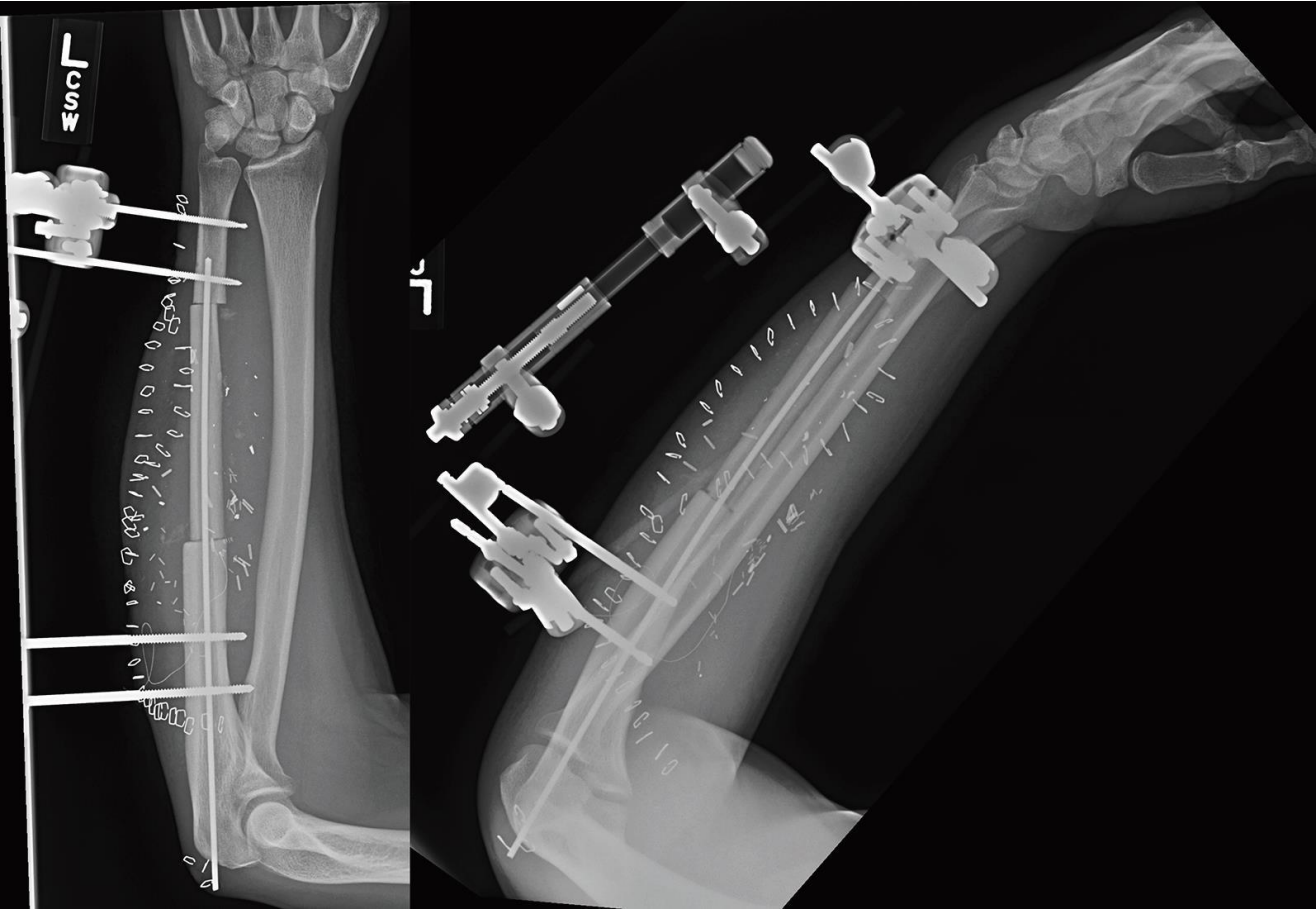
Interposition placement of vascularized fibula into bone defect, stabilized with Stryker Monotube Triax External Fixation System and flexible ulnar nail.

**Figure 4:**
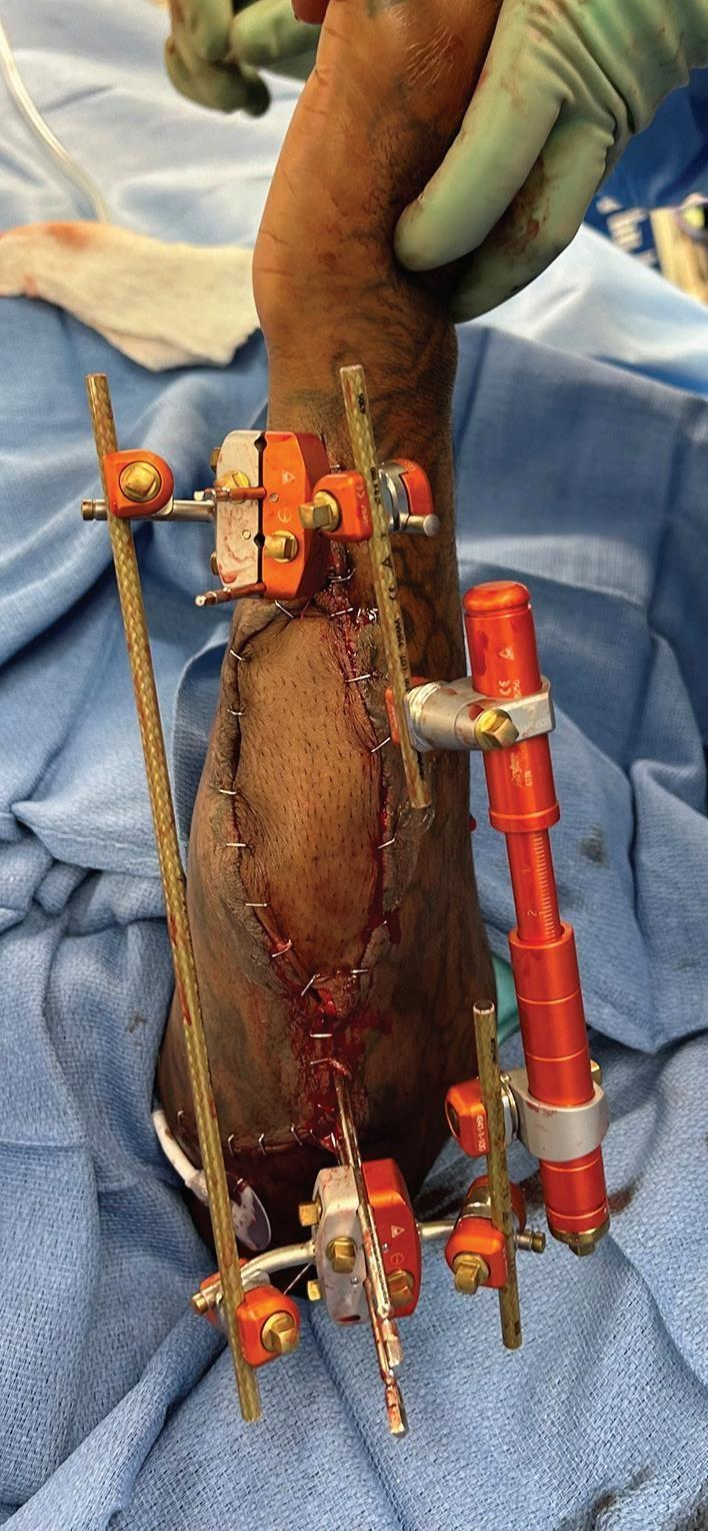
Skin paddle inset provided soft tissue coverage.

On Day 15, the patient underwent skin grafting of the donor site and was discharged from the hospital on Day 22 following completion of standard prophylactic antibiotics and removal of the skin graft bolster, with prophylactic Lovenox and aspirin 81 mg.

The patient underwent removal of the external fixator and ulnar nail with definitive internal fixation 6 weeks postoperatively and returned to full duty work as a deliveryman 3 months after injury. At 12 months, he demonstrated complete soft tissue healing and radiographic consolidation (Figures 5 and 6). He demonstrated full elbow flexion and extension, as well as functional pronosupination arc > 100°. He had full return of sensation in the ulnar distribution as well as 5/5 intrinsic strength and was able to return to jogging and lifting weights with expected deconditioning, but no pain or deficits otherwise.

**Figure 5:**
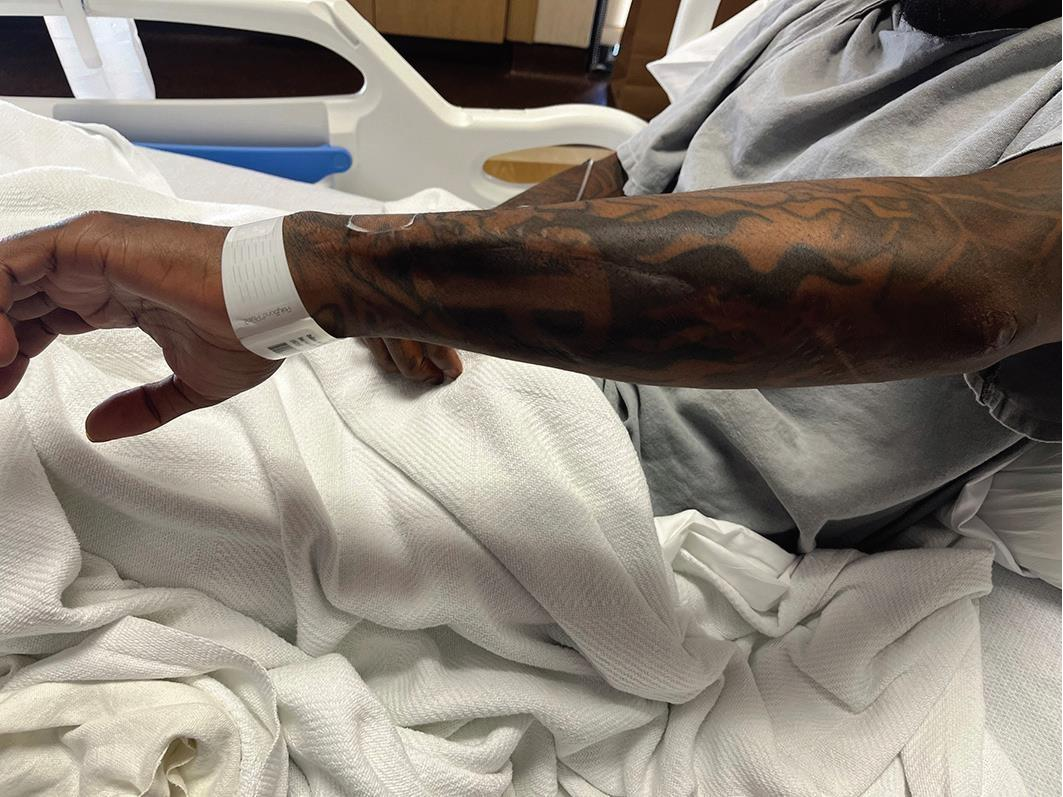
Complete healing of soft tissues at 12‐month follow‐up.

**Figure 6:**
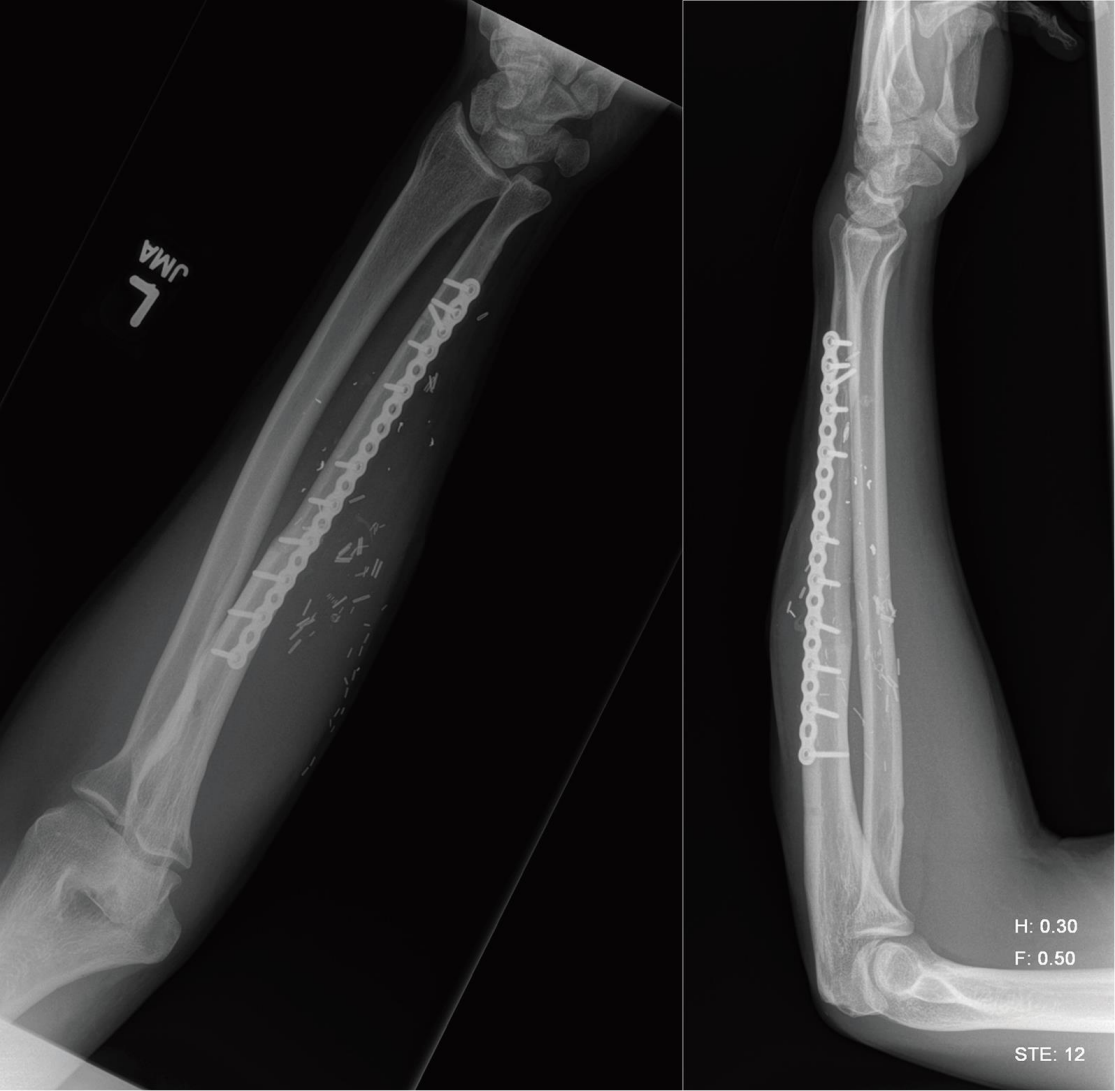
Complete radiographic consolidation of vascularized fibula at 12 months.

### 2.2. Case 2

A 21‐year‐old right‐handed male with no significant medical or surgical history was transferred 3 days following a dune buggy accident after undergoing exploratory laparotomy and bladder repair in the Caribbean. He sustained an open left intra‐articular distal humerus fracture‐dislocation with associated coronoid process fracture, medial collateral ligament tear, and radial nerve neuropraxia manifesting with inability to extend the wrist. He did otherwise have a well‐perfused extremity with palpable pulses and otherwise normal findings on median and ulnar nerve testing. His soft tissue injury included two large elbow wounds grossly contaminated with debris and purulence: a medial wound extending proximally and a nearly circumferential posterolateral wound (Figure 7).

**Figure 7:**
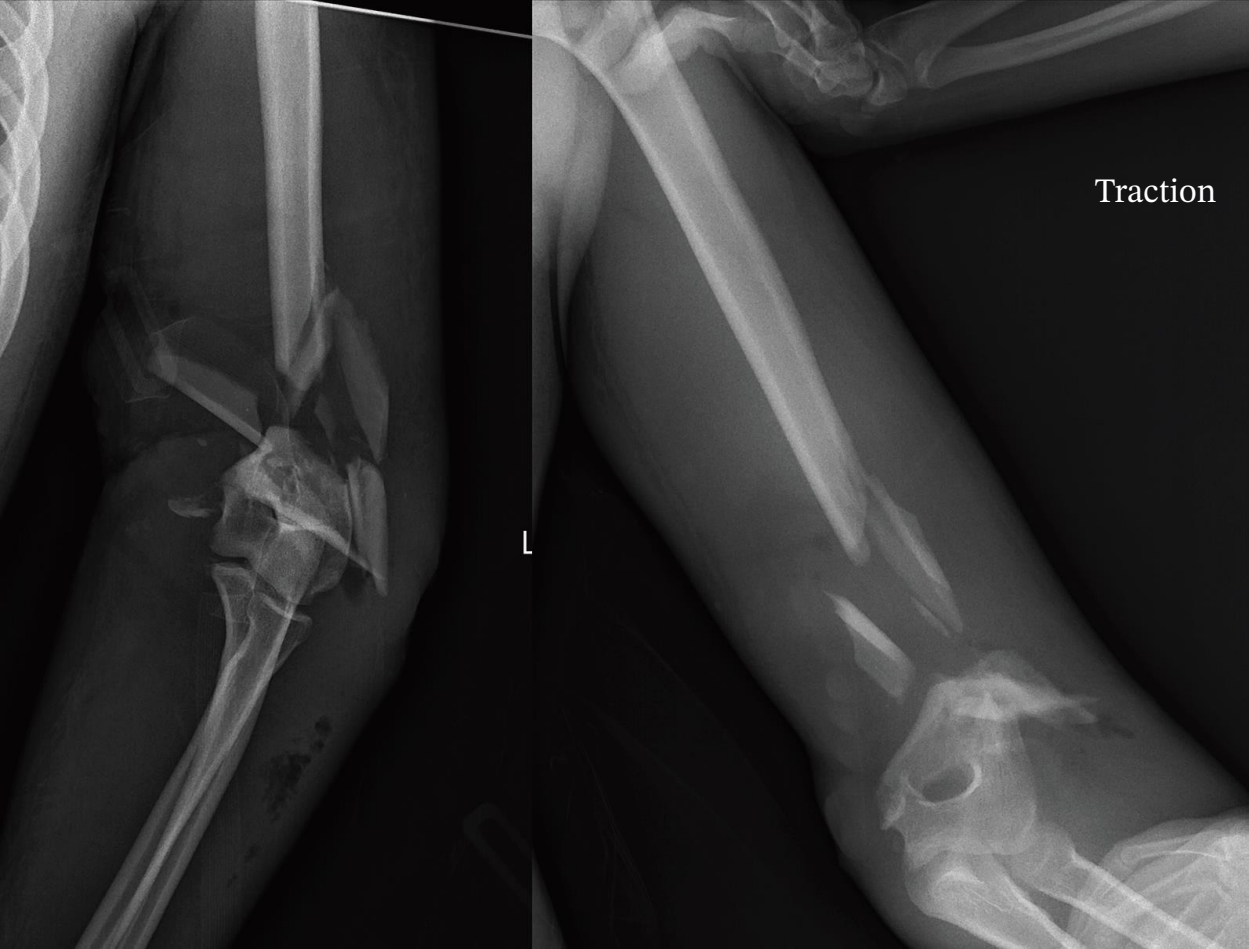
Comminuted intraarticular distal humerus fracture with numerous large fragments of devitalized bone.

Initial exploration revealed large segment of devascularized distal humeral shaft devoid of periosteum, but identified intact radial and ulnar nerves. Initial bone cultures showed growth of *Enterococcus durans* and rare *Pseudomonas aeruginosa*, empirically treated with meropenem, vancomycin, and micafungin. The patient underwent serial debridement, placement of antibiotic spacer, and temporary skeletal fixation until the wound was clean of all purulence and returned with negative cultures. Final wound measurements were 18 × 16 cm medially, 15 × 10 cm laterally and final bone deficit of 8.5 cm (Figure 8).

**Figure 8:**
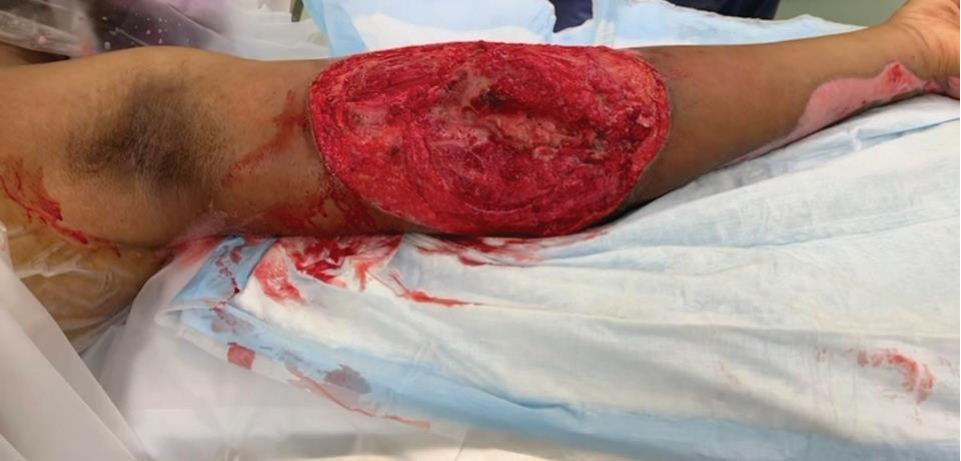
Soft tissue defects measured 18 × 16 cm medially, 15 × 10 cm laterally.

On hospital Day 18 the patient underwent VFG reconstruction with an 8 × 5 cm skin paddle. A lateral column distal humerus plate was provisionally fixed and microanastomosis was carried out with 4.0 venous coupler of peroneal vein to cephalic vein, and end‐to‐side anastomosis of peroneal artery to brachial artery. The VFG was press‐fit into the medial column of the distal humerus and fixated through a lateral distal humerus plate (Figure 9). Soft tissue coverage of all vital structures, graft, and hardware was achieved with the skin paddle. On Day 25, he underwent adjacent tissue transfer and skin grafting and was discharged the following day with 7 days of oral clindamycin and levofloxacin based on culture sensitivities, as well as prophylactic Lovenox and aspirin 81 mg.

**Figure 9:**
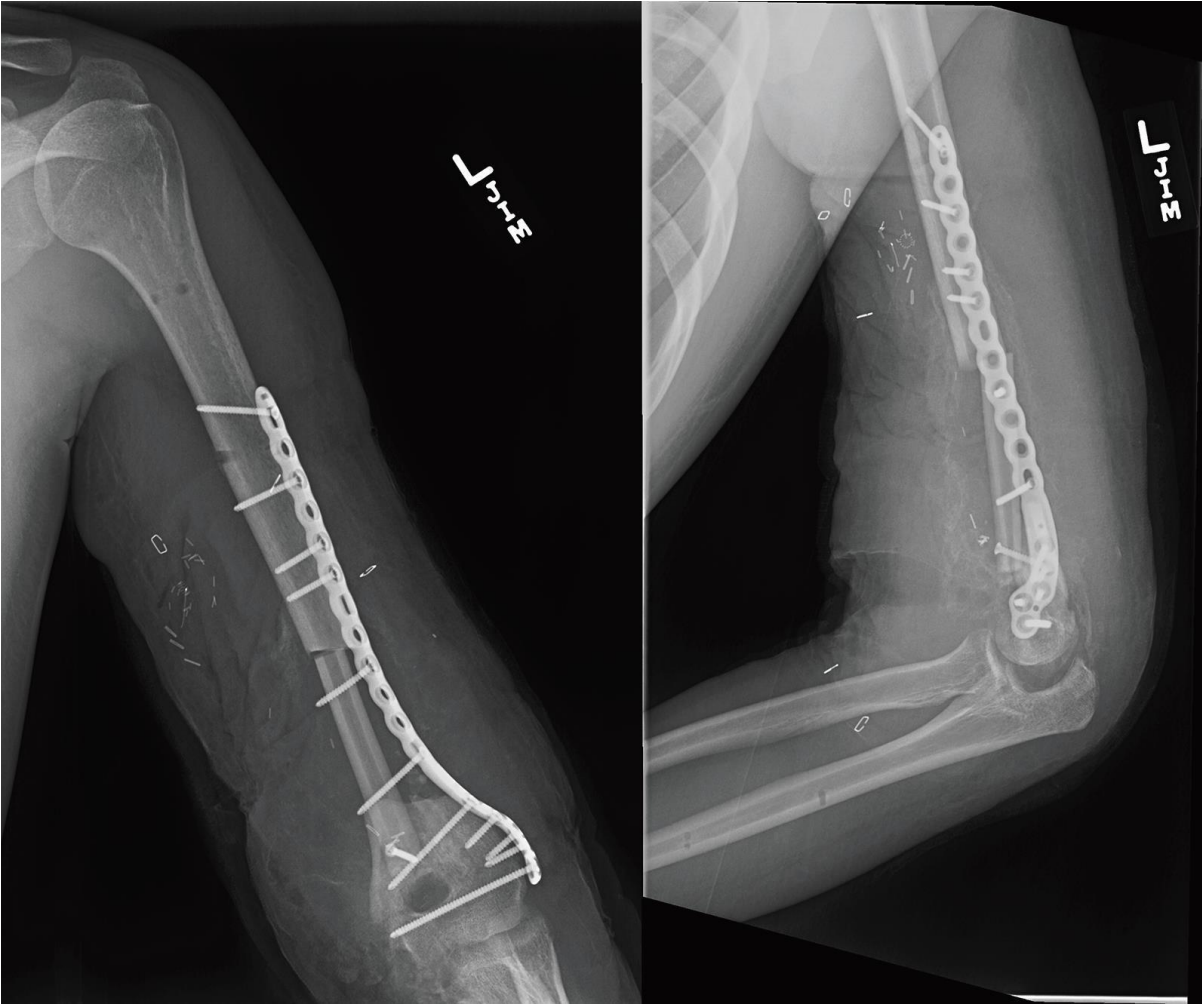
Open reduction internal fixation of distal humerus fracture with lateral distal humerus plate and press‐fit placement of vascularized fibula into medial column to replace 8.5 cm defect.

The patient returned to work as a traveling musician at 5 months with the ability to independently carry heavy equipment. At 8 months follow‐up, the patient had full return of wrist extension, elbow range of motion 90–150 degrees, complete healing of soft tissues, and bridging bone formation (Figures 10 and 11). He returned to jogging and at 9 months resumed weightlifting with expected deconditioning, but no deficits. At 14 months, he continued to improve in strength and range of motion without complication.

**Figure 10:**
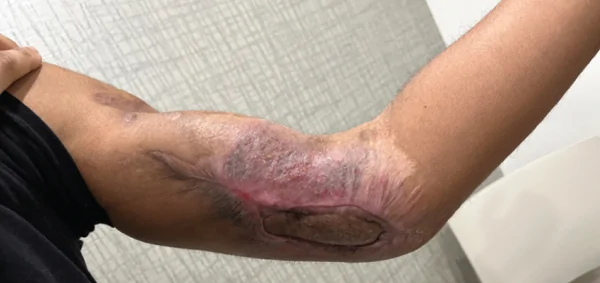
Healing of open left distal humerus fracture 8 months post‐operatively following vascularized osteocutaneous fibular flap and split thickness skin grafting.

**Figure 11:**
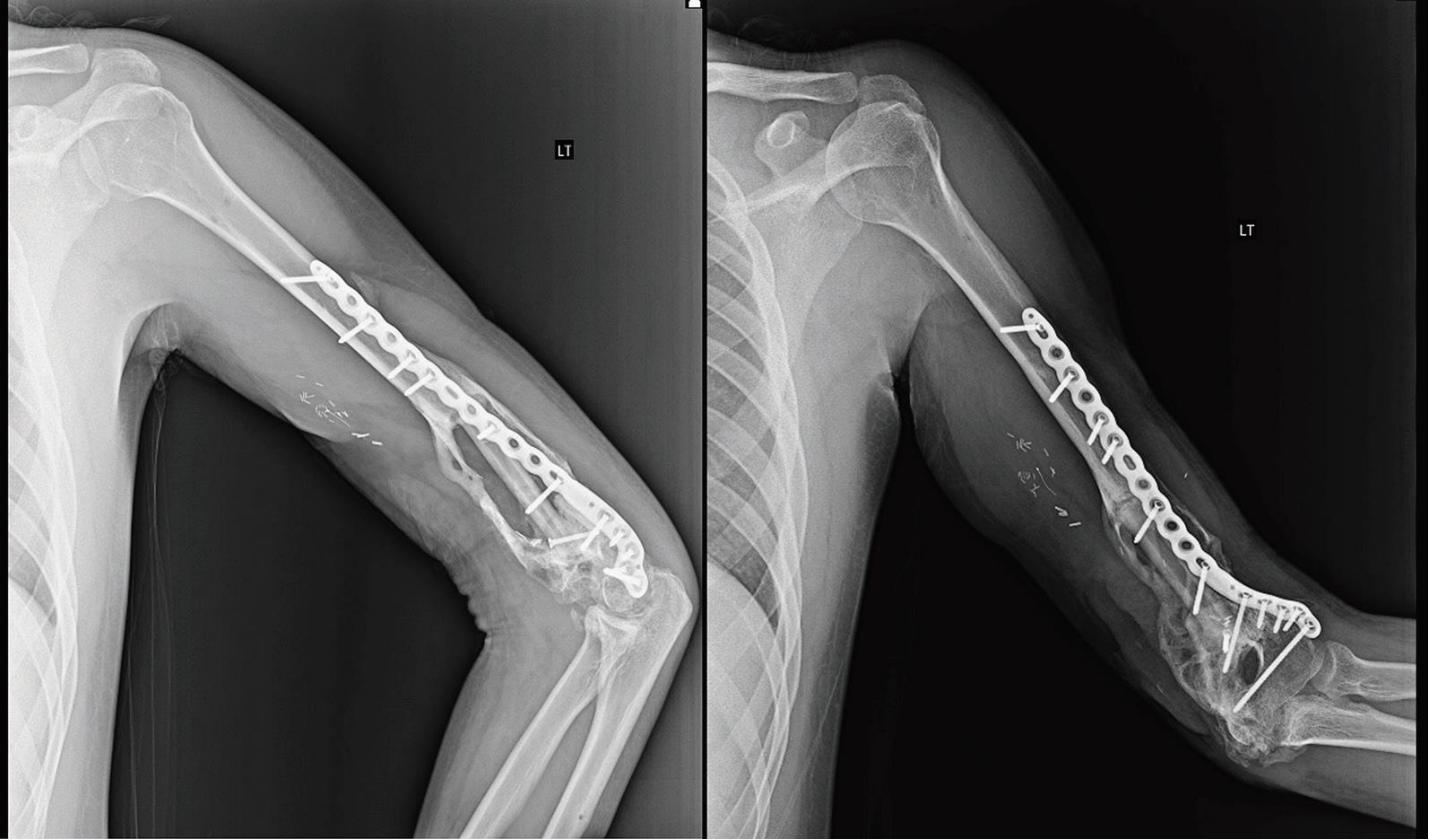
Stable construct at 8‐month follow‐up. Bridging bone formation demonstrates ongoing vascularity of the fibular graft.

## 3. Discussion

Critical bone loss in the traumatic upper extremity is uncommon [1]. Less commonly described in literature is the use of VFG as initial definitive treatment. In a systematic review, Liaw et al. identified eight studies with a median length of time from injury to reconstruction of 15 months [11]. Our cases were performed at 6 and 21 days postinjury and both patients returned to unrestricted heavy duty labor within 5 months of injury.

In the first case, we stabilized the VFG with an external fixator and flexible ulnar nail prior to exchange for internal fixation in order to limit possibility of infection that could compromise the graft. Following internal fixation, the patient rapidly progressed and returned to heavy‐duty labor within 3 months of injury. The second case had a delayed presentation with a heavily contaminated field. This required multiple rounds of debridement to achieve a clean field with negative cultures before we felt comfortable implanting definitive hardware; otherwise, reconstruction with VFG could have been performed earlier. Unfortunately with a distal humerus fracture, external fixation would not have provided sufficient stability to achieve length, rotation, and alignment with the VFG as an interposition graft.

Induced membrane formation has equivalent union rates compared with the VFG, but may have longer time to union depending on defect size and location [15]. Bone grafting is performed 6–8 weeks after the first stage, but risks failure of membrane formation or graft resorption [1, 16]. Distraction osteogenesis occurs at 1 mm a day following a 1 week latency period—which would be 3 months in our patients—followed by a consolidation phase of up to twice that period while requiring diligent pin care and compliance [1, 17].

VFG allows soft tissue coverage, earlier fixation and early activity. This aligns with current dogma for early microsurgical intervention to decrease length of hospitalization and cost [13]. This technique has been used for early management of critical bone loss in lower extremity trauma with excellent results in terms of flap survival; [18] however there are risks of mechanical complications or failure of the fibular flaps as a result of weight‐bearing [12, 18]. This risk is much lower in the upper extremity as our patients were able to offload their operative extremities during recovery and progressed quickly to full weight‐bearing without complication. Furthermore, the discrepancy in cross‐sectional diameter between the VFG and ulnar shaft in our first patient was minimal [19]; which likely contributed to less time to hypertrophy and consolidation for earlier weight‐bearing.

The hospital stays of 26 and 22 days are comparable to reports in literature [5]. Furthermore, our operations were performed during index hospitalization whereas previous reports were performed at subsequent or ongoing hospitalizations [5, 6, 11, 12]. By 12 months both patients demonstrated appropriate soft tissue and bony healing, consistent with timelines reported in literature for nonacute reconstruction [5–7, 12]. Neither patient had experienced any donor site morbidity other than expected deconditioning. Both patients were highly satisfied with their results and ability to return to their normal life in an expedited manner.

### 3.1. Limitations

These cases were both investigated retrospectively, so we are limited in our comparison with more traditional techniques for achieving osteosynthesis given critical bone loss in the upper extremity. In both these cases, the patients had residences in the area but traveled frequently out of state. As a result, although we were able to follow‐up with the patients more than 1 year postoperatively, we were unable to establish follow‐up at desired, regular intervals. This limited our ability to determine precise timing to radiologic union and complete soft tissue healing. Literature reports median time to consolidation in the upper extremity at 4 months [11], and it is unknown whether our patients had union at the time; though they did report at later follow‐up that they had returned to full duty by 3 months and 5 months, respectively. Subsequent radiographs demonstrated graft consolidation and no mechanical complications.

We stress that previous reports and descriptions in literature for the upper extremity tended to occur in a delayed fashion [5, 11, 12], but we believe more consideration should be given for early, if not immediate, use of the VFG especially when soft tissue and bony reconstruction is needed. Preoperatively, the vascular status of the patient′s afflicted extremity should be thoroughly evaluated to determine the dominant inflow for the hand, assuring that a vascularized bone graft would not compromise the extremity. When used for humerus reconstruction, an end‐to‐side anastomosis to the brachial artery would be used. The use of VFG does require specialized microsurgical techniques; however, is of immense benefit to the patient so multidisciplinary involvement of orthopedic and microvascular reconstructive surgeons should be obtained early in the patient′s care.

## 4. Conclusion

The VFG is an underutilized option for acute, definitive reconstruction of upper extremity trauma with critical bone loss. This procedure can address bone and soft tissue deficits in a single‐stage with a rapid timeline for full recovery.

## Funding

No funding was received for this manuscript.

## Conflicts of Interest

The authors have nothing to report.

## Data Availability

The data that support the findings of this study are available on request from the corresponding author. The data are not publicly available due to privacy or ethical restrictions.
